# Allergy reduces the risk of meningioma: a meta-analysis

**DOI:** 10.1038/srep40333

**Published:** 2017-01-10

**Authors:** Peng-fei Wang, Wen-Jun Ji, Xiao-hui Zhang, Shou-wei Li, Chang-Xiang Yan

**Affiliations:** 1 Department of Neurosurgery, Sanbo Brain Hospital, Capital Medical University, China; 2 Department of Interventional Neuroradiology, Beijing Neurosurgical Institute, Beijing Tiantan Hospital, Capital Medical University, Beijing, China; 3 Department of Rheumatology and Clinical Immunology, Peking University First Hospital, Beijing, China.

## Abstract

Meningiomas are the most common brain tumours; however, little is known regarding their aetiology. The data are inconsistent concerning atopic disease and the risk of developing meningioma. Thus, we conducted a meta-analysis to investigate the association between allergic conditions and the risk of developing meningioma. A systematic literature search was conducted using PubMed and Web of SCI from Jan 1979 to Feb 2016. Two investigators independently selected the relevant articles according to the inclusion criteria. Eight case-control studies and 2 cohort studies were included in the final analysis, comprising 5,679 meningioma cases and 55,621 control subjects. Compared with no history of allergy, the pooled odds ratio (OR) for allergic conditions was 0.81 (0.70–0.94) for meningioma in a random-effects meta-analysis. Inverse correlations of meningioma occurrence were also identified for asthma and eczema, in which the pooled ORs were 0.78 (0.70–0.86) and 0.78 (0.69–0.87), respectively. A reduced risk of meningioma occurrence was identified in hay fever; however, the association was weak (0.88, 95% CI = 0.78–0.99). The source of this heterogeneity could be the various confounding variables in individual studies. Overall, the current meta-analysis indicated that allergy reduced the risk of developing meningiomas. Large cohort studies are required to investigate this relationship.

Meningiomas are the most frequently reported brain tumours and account for 36.4% of all central nervous system (CNS) tumours. The incidence of meningioma increases with age and is 2.5 times higher in females than in males^1^ Approximately 98.7% of meningiomas are reported as benign tumours and are classified as grade I according to the 2007 World Health Organization (WHO) grading system^1^,^2^. Benign tumours are associated with improved patient survival; however, only 33% of meningioma patients exhibited no neurological deficits in a long-term follow-up^3^. This unfavourable prognosis necessitates the need to develop potential preventive strategies. Unfortunately, a limited number of factors associated with the development of meningioma have been identified, including exposure to ionizing radiation, high body mass index (BMI) and a low level of physical activity^4^,^5^.

Numerous studies have investigated the relationship between the occurrence of brain tumours and allergic conditions, including asthma, eczema, and hay fever. Atopic diseases have been inversely correlated with the risk of developing gliomas^6^,^7^,^8^,^9^. However, there are no consistent findings that link meningiomas and atopic diseases, with the exception of eczema^7^,^10^,^11^,^12^,^13^. In the two previous meta-analysis studies of atopy and the risk of meningioma development, no significant correlation between allergy history and meningioma was identified^6^,^14^. However, recent studies have suggested a strong inverse association between allergy history (including allergy, asthma, eczema and hay fever) and meningiomas^8^,^15^,^16^. Consequently, a meta-analysis was performed in the current study to address these conflicting results.

## Results

### Identification of relevant studies

Eleven articles, including 9 case-control studies and 3 cohort studies, that investigated the relationship between brain meningiomas and allergic conditions were identified through literature searches^8^,^10^,^11^,^12^,^13^,^15^,^16^,^17^,^18^,^19^. There were three cohorts in Schwartzbaum’s study, but one was excluded due to expansive exposure definitions^11^. One case-control study^19^ was removed because the data were included in a larger investigation^17^. Finally, 5,679 patients with meningiomas and 55,621 control subjects were included in this meta-analysis. Two case-control studies conducted in southeast England shared a dataset that comprised 225 patient cases and 630 control subjects. However, the shared dataset was comparatively smaller than the total dataset (4% and 1% of the total, respectively), and we did not exclude the data as was done in previous studies^14^.

### Characteristics of the included studies

The details of the included subjects are presented in Table 1. All investigations were conducted in countries with a relatively high socioeconomic status, including Australia, New Zealand, Israel, North America and Europe, from 1977 to 2010. All meningioma cases were medically diagnosed. The odds ratio (OR) and 95% confidence interval (CI) provided by each study were adequately adjusted according to age and gender, and some values were controlled based on region and socioeconomic status (Table 2). An assessment of the studies included using the Newcastle Ottawa Scale (NOS) is presented in the supplementary files.

### Meta-analysis of allergy and meningioma

**Figure 1:**
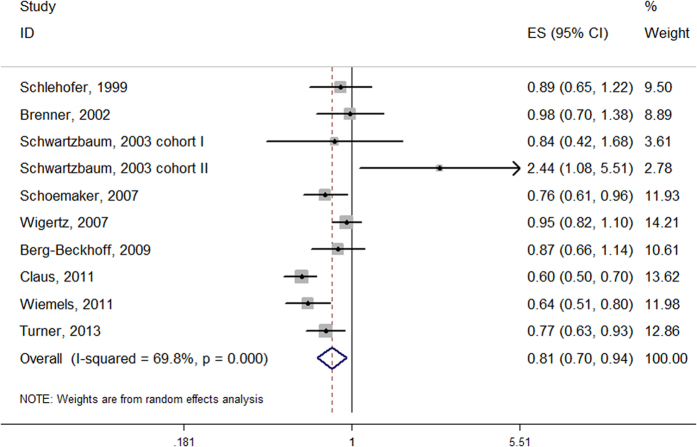
Meta-analysis of the association between any allergy and risk of developing meningiomas.

**Figure 2:**
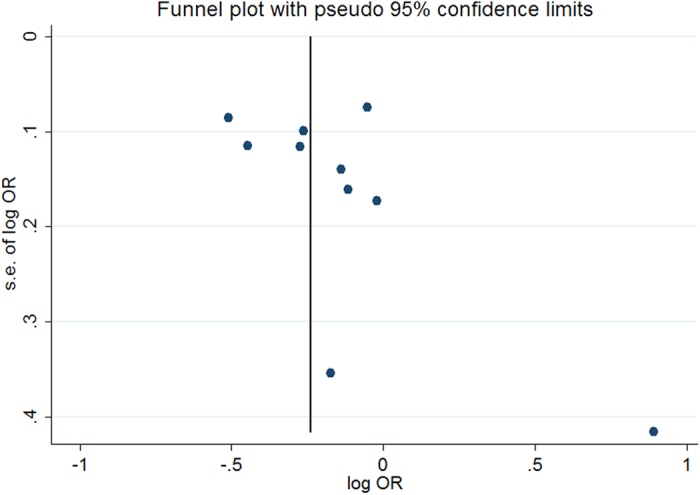
Funnel plots of publication test for allergy with the risk of meningioma occurrence.

Study-specific log ORs and the corresponding confounders adjusted are shown in Table 2. A patient history of allergic conditions was associated with a lower risk of meningiomas in a random model (Pooled OR = 0.81, 95% CI = 0.70–0.94, Fig. 1). A substantial degree of heterogeneity was identified in the included studies (I^2^ = 69.8%, P = 0.000). We identified an inverse association in the case-control study (Pooled OR = 0.79, 95% CI = 0.68–0.90); however, we failed to identify similar results in the cohort study (Pooled RR = 1.40, 95% CI = 0.49–3.98, Table 3). Moreover, the heterogeneity was not significantly reduced after stratification by study design, proxy rates or other characteristics. The exception to this finding was stratification by the adjusted cofounders (age and gender vs. age, gender, region, socioeconomic status and other factors), which demonstrated a reduction in heterogeneity in the later studies (I^2^ = 44.1%) compared with the alternative (I^2^ = 82.6%). Proxy respondents were less likely to report an allergic condition, which may lead to bias^11^. A relatively high ratio of proxy respondents was apparent in two studies^10^,^17^, which were excluded in the further analyses. An inverse correlation of allergy occurrence with meningiomas persisted (Pooled OR = 0.79, 95% CI = 0.67–0.94). No publication bias was identified among the studies according to the Begg or Egger tests (p = 0.152 and 0.278, respectively). As shown in the Funnel plots, most data points were within the funnel area for the risk of allergy with meningioma (Fig. 2).

### Specific allergic conditions and meningiomas

The relationship between the risk of developing meningioma and the occurrence of specific allergic conditions was also investigated in 8 asthma studies, 9 eczema studies and 6 hay fever studies (Table 2). We identified an inverse relationship between asthma occurrence and the risk of developing meningiomas (Pooled OR = 0.78, 95% CI = 0.70–0.86), based on 16,491 participants, including 5,444 participants diagnosed with meningioma. A total of 5,424 cases and 25,658 controls were included in the study of eczema, which was inversely correlated with occurrence of meningioma (Pooled OR = 0.78, 95% CI = 0.69–0.87). There were 2,982 subjects with a diagnosis of meningioma and 23,096 control subjects included in the study, which indicates a lower margin of meningiomas (Pooled OR = 0.88, 95% CI = 0.78–0.99). No heterogeneity was identified in this analysis (Table 3).

## Discussion

In the two previous meta-analyses, no relationship between allergy history and meningioma occurrence was identified^6^,^14^. However, these findings were challenged by the inclusion of recently published data that demonstrated a significant inverse relationship between the two conditions^8^,^15^,^16^. Our results indicated an inverse association between allergic conditions and meningiomas, with the inclusion of 5,679 meningioma cases and 55,621 controls. The association was also strong in the patients with asthma and eczema. We initially determined that hay fever was correlated with a reduced risk of meningiomas; however, this correlation was not obvious.

Our results were also supported by the fact that serum IgE levels (immunoglobulin E, a biomarker of atopic allergy) were reduced in patients with meningiomas compared with control subjects^15^. Most importantly, IgE was likely to be an explanation for the mechanism behind allergy protected meningioma occurrence. Allergy mediated by IgE played a critical role in malignancies in the field of AllergoOncology^20^. It was well observed that IgE acted superiorly to any immunoglobulins in targeting overexpressed tumor antigens, suggesting a positive role in natural immune surveillance. Furthermore, a hyper-reactive immune state system was more capable of recognizing and killing potentially cancerous cells^21^,^22^. Moreover, macrophages, eosinophils and mast cells armed with IgE could become potent effectors in antitumor immunity^20^,^23^,^24^. Additionally, reverse causality was possibly another explanation for the protective role of allergy. And this meant that the tumor itself suppressed immune function^25^. Compared with benign meningiomas, aggressive meningiomas always exhibited immunological defects such as a decrease in CD4^+^ and CD8^+^ T lymphocytes, an increase in FOXP3+ Treg and PD-L1 (programmed death-ligand 1) levels^26^,^27^,^28^,^29^,^30^.

There were several limitations in our study. First, there was substantial heterogeneity in the analysis of meningiomas with allergy, i.e., 69.8% by I^2^. The occurrence of meningiomas was likely to vary according to region, race, smoking history and socioeconomic status^1^,^31^,^32^,^33^, which were considered in some of the included studies when calculating the log ORs. Following the stratification of adjusted confounders, the heterogeneity was reduced to 44.1% by I^2^, which provided log ORs adjusted by at least 3 compounding variables (e.g., age, gender, and region). Moreover, the relationships between ionizing radiation exposure, BMI and physical activity and the risk of meningiomas were completely ignored in the included studies^4^,^5^. This exclusion may provide one potential source of heterogeneity. Second, our analysis was mainly composed of case-control studies, which are sensitive to recall bias. However, the cohort studies included in the analysis consisted of small populations of cases and controls, which comprised 69 and 44,048 individuals, respectively. The results in the cohort studies were also completely different; however, the studies were conducted with very similar methods^11^. Large cohort studies are necessary to confirm the inverse relationship. Third, all studies included in the final analysis were conducted in Europe, North America, Australia, New Zealand and Israel, countries with prosperous economies and advanced medicine. The conclusions outlined in the present study may not be applicable to other regions. In addition, publication bias was also a concern. Unpublished data demonstrating that allergic conditions are not correlated with meningiomas may exist. This missing information is likely to weaken to correlation of allergy with the risk of meningiomas.

In summary, allergic conditions were significantly associated with a reduced risk of developing meningiomas in this meta-analysis. We also identified a lower risk of meningiomas in subjects with asthma and eczema, whereas hay fever was weakly correlated with meningioma occurrence. Large cohort studies that include adequate samples remain necessary to investigate this relationship.

## Methods

### Literature search and study selection

Two authors independently conducted literature searches using PubMed and ISI Wed of SCI from Jan 1979 to Feb 2016, using the search terms brain tumour, meningioma, allergy, atopy, asthma, eczema, and hay fever. The eligibility criteria for the studies included in this meta-analysis were as follows: (1) contained data from a case-control study or a cohort study, (2) a medical diagnosis of meningioma, (3) investigated the relationship between the risk of meningioma and allergy, and (4) provided an OR or relative risk (RR) with estimates of 95% CIs (or data to calculate CIs). In cases in which patients were included in more than one publication, the most recent study was selected for analysis.

### Data extraction and assessment of study quality

All potential articles were independently reviewed and the data were extracted by two investigators; the variables included the first author’s name, publication year, geographical origin, number of cases and controls, exposure definitions and risk estimates. Discrepancies were solved by discussion and consensus. We assessed the study quality using the NOS^34^. Nine of the 10 included studies were awarded seven stars, whereas Brenner’s studies were awarded six stars.

### Statistical analysis

To compute the pooled ORs of allergy in meningiomas, we used the multivariate-adjusted risk estimates in each study rather than raw data. Study-specific log RRs or log ORs were weighted by the inverse of their variances. We present the pooled ORs in the random-effect models, which provide pooled results that are less precise but provide correct coverage. The heterogeneity among the studies was assessed by the method of DerSimonian and Laird Q^35^. We quantified the heterogeneity using I^2^. To investigate the potential origin of the heterogeneity, the included studies were further divided into subgroups according to the study characteristics (case-control vs. cohort study), proxy rates (high vs. low), and potential cofounders adjusted in individual studies (age, gender vs. age, gender, region, social-economics states and other factors). Publication bias was assessed by Funnel plots, the Begg test, and the Egger test.

## Additional Information

**How to cite this article**: Wang, P.-f. *et al*. Allergy reduces the risk of meningioma: a meta-analysis. *Sci. Rep.* **7**, 40333; doi: 10.1038/srep40333 (2017).

**Publisher's note:** Springer Nature remains neutral with regard to jurisdictional claims in published maps and institutional affiliations.

## Supplementary Material

Supplement Material

## Figures and Tables

**Table 1:** Description of included studies regarding allergic status and risk of meningioma development.

First author, date (reference)	Country	Design	Case/control	Type of control (% response rate)	% Proxy reporting of case (control)	Exposure assessment
Schlehofer^17^	Six countries	Case-control	331/1123	Population (not specified)	3.0% (7.8%)	Interview and SEARCH questionnaire
Brenner^10^	US	Case-control	193/777	Non-cancer hospital	11% (4%)	Interview and physician diagnosis
Schwartzbaum^11^	Sweden	Cohort I	41/14493	Twins born 1886–1925	0	E-mail questionnaire
		Cohort II	28/29555	Twins born 1926–1958	0	E-mail questionnaire
Schoemaker^12^	UK	Case-control	475/1716	Population (57%)	0	Interview
Wigertz^13^	Five countries	Case-control	1210/3309	Population (50%)	0.1% (2%)	Interview; questionnaire
Berg-Beckhoff^18^	Germany	Case-control	380/762	Population (62.7%)	0.3% (0.3%)	Computer-assisted personal interview
Claus^16^	US	Case-control	1124/1000	Population (74%)	0 (0)	Interview; questionnaire
Wiemels^15^	US	Case-control	1065/634	Population (54%)	0 (0)	Interview; questionnaire
Turner^8^	Five countries	Case-control	832/2252	Population (not specified)	2% (0.4%)	Computer-assisted personal interview

**Table 2:** Pooled ORs with 95% Cls for allergy and meningioma.

RRs (95% Cl) for history of					
First author, date (reference)	Any allergy	Asthma	Eczema	Hay fever	Potential confounders adjusted in the analysis
Schlehofer^17^	0.89 (0.65–1.22)	0.82 (0.46–1.44)	0.68 (0.42–1.08)		age (5-year group), gender, and region
Brenner^10^	0.98 (0.70–1.38)	0.86 (0.53–1.40)	0.80 (0.42–1.53)	0.93 (0.58–1.50)	age, gender, race, and region
Schwartzbaum^11^	0.84 (0.42–1.68)		0.72 (0.30–1.70)	0.85 (0.33–2.17)	age and gender
	2.44 (1.08–5.51)				
Schoemaker^12^	0.76 (0.61–0.96)	0.85 (0.61–1.18)	0.72 (0.51–1.02)	0.81 (0.62–1.06)	age (5-year group), gender, region, interview year, and SES (Townsend index)
Wigertz^13^	0.95 (0.82–1.10)	0.94 (0.74–1.20)	0.74 (0.60–0.91)	0.93 (0.77–1.12)	age, gender, education, country, and region
Berg-Beckhoff^18^	0.87 (0.66–1.14)	0.78 (0.47–1.28)	0.84 (0.61–1.15)	0.98 (0.67–1.39)	age, gender, socioeconomic status, region, and smoking
Claus^16^	0.6 (0.5–0.7)	0.7 (0.6–0.9)	0.8 (0.6–1.1)		age and gender
Wiemels^15^	0.64 (0.51–0.80)	0. 65 (0.50–0.86)	0.95 (0.67–1.34)		age, gender, smoking, race and education
Turner^8^	0.77 (0.63–0.93)	0.78 (0.59–1.03)	0.74 (0.56–0.98)	0.80 (0.63–1.01)	age (5-year group), gender, region, country and education

**Table 3:** Pooled odd ratios (ORs) and 95% confidence intervals (CIs) of meningiomas in the subgroup analysis.

Factor	No. of studies	No. of cases	No. of controls	Pooled ORs (95% CI)_random effects_	I^2^	P_heterogeneity_
All studies	10	5,679	55,621	0.81 (0.70–0.94)	69.80%	0.000
Case-control studies	8	5,610	11,573	0.79 (0.68–0.90)	68.60%	0.002
Cohort studies	2	69	44,048	1.40 (0.49–3.98)	73.80%	0.051
Adjusted cofounders (age and gender)	3	1193	45,048	0.99 (0.46–2.14)	82.60%	0.003
Adjusted cofounders (e.g., age, gender, and region)	7	4,486	10,573	0.82 (0.73–0.93)	44.10%	0.097
Lower proxy rate	2	5,155	53,721	0.79 (0.67–0.94)	74.50%	0.000
Higher proxy rate	8	524	1900	0.93 (0.74–1.17)	0.00%	0.683
Asthma	8	5,444	11,047	0.78 (0.70–0.86)	0.00%	0.593
Eczema	9	5,424	20,234	0.78 (0.69–0.87)	0.00%	0.966
Hay fever	6	2,982	20,114	0.88 (0.78–0.99)	0.00%	0.887

## References

[b1] Ostrom Q. T. . CBTRUS Statistical Report: Primary Brain and Central Nervous System Tumors Diagnosed in the United States in 2008–2012. Neuro-oncology 17 Suppl 4, iv1–iv62, doi: 10.1093/neuonc/nov189 (2015). 26511214 PMC4623240

[b2] Louis D. N. . The 2007 WHO classification of tumours of the central nervous system. Acta neuropathologica 114, 97–109, doi: 10.1007/s00401-007-0243-4 (2007). 17618441 PMC1929165

[b3] van Alkemade H. . Impaired survival and long-term neurological problems in benign meningioma. Neuro-oncology 14, 658–666, doi: 10.1093/neuonc/nos013 (2012). 22406926 PMC3337301

[b4] Niedermaier T. . Body mass index, physical activity, and risk of adult meningioma and glioma: A meta-analysis. Neurology 85, 1342–1350, doi: 10.1212/WNL.0000000000002020 (2015). 26377253

[b5] Wiemels J., Wrensch M. & Claus E. B. Epidemiology and etiology of meningioma. Journal of neuro-oncology 99, 307–314, doi: 10.1007/s11060-010-0386-3 (2010). 20821343 PMC2945461

[b6] Linos E., Raine T., Alonso A. & Michaud D. Atopy and risk of brain tumors: a meta-analysis. Journal of the National Cancer Institute 99, 1544–1550, doi: 10.1093/jnci/djm170 (2007). 17925535

[b7] Deckert S., Kopkow C. & Schmitt J. Nonallergic comorbidities of atopic eczema: an overview of systematic reviews. Allergy 69, 37–45, doi: 10.1111/all.12246 (2014). 24053642

[b8] Turner M. C. . Allergy and brain tumors in the INTERPHONE study: pooled results from Australia, Canada, France, Israel, and New Zealand. Cancer Causes Control 24, 949–960, doi: 10.1007/s10552-013-0171-7 (2013). 23443320

[b9] Amirian E. S. . Approaching a Scientific Consensus on the Association between Allergies and Glioma Risk: A Report from the Glioma International Case-Control Study. Cancer epidemiology, biomarkers & prevention: a publication of the American Association for Cancer Research, cosponsored by the American Society of Preventive Oncology 25, 282–290, doi: 10.1158/1055-9965.EPI-15-0847 (2016). PMC4874516 26908595

[b10] Brenner A. V. . History of allergies and autoimmune diseases and risk of brain tumors in adults. International journal of cancer. Journal international du cancer 99, 252–259, doi: 10.1002/ijc.10320 (2002). 11979441

[b11] Schwartzbaum J. . Cohort studies of association between self-reported allergic conditions, immune-related diagnoses and glioma and meningioma risk. International journal of cancer. Journal international du cancer 106, 423–428, doi: 10.1002/ijc.11230 (2003). 12845684

[b12] Schoemaker M. J. . History of allergic disease and risk of meningioma. Am J Epidemiol 165, 477–485, doi: 10.1093/aje/kwk048 (2007). 17182979

[b13] Wigertz A. . Allergic conditions and brain tumor risk. Am J Epidemiol 166, 941–950, doi: 10.1093/aje/kwm203 (2007). 17646205

[b14] Wang M. . Inverse association between eczema and meningioma: a meta-analysis. Cancer Causes Control 22, 1355–1363, doi: 10.1007/s10552-011-9808-6 (2011). 21710191

[b15] Wiemels J. L. . Reduced allergy and immunoglobulin E among adults with intracranial meningioma compared to controls. International journal of cancer. Journal international du cancer 129, 1932–1939, doi: 10.1002/ijc.25858 (2011). 21520030 PMC3337969

[b16] Claus E. B. . Family and personal medical history and risk of meningioma. Journal of neurosurgery 115, 1072–1077, doi: 10.3171/2011.6.JNS11129 (2011). 21780859 PMC3241000

[b17] Schlehofer B. . Role of medical history in brain tumour development. Results from the international adult brain tumour study. International journal of cancer. Journal international du cancer 82, 155–160 (1999). 10389745 10.1002/(sici)1097-0215(19990719)82:2<155::aid-ijc1>3.0.co;2-p

[b18] Berg-Beckhoff G. . History of allergic disease and epilepsy and risk of glioma and meningioma (INTERPHONE study group, Germany). Eur J Epidemiol 24, 433–440, doi: 10.1007/s10654-009-9355-6 (2009). 19484497

[b19] Ryan P., Lee M. W., North B. & Mcmichael A. J., Ryan P., Lee M. W., North J. B. & McMichael A. J. Risk factors for tumors of the brain and meninges: results from the Adelaide Adult Brain Tumor Study. Int J Cancer 51. International Journal of Cancer 51, 20–27 (1992). 1563840 10.1002/ijc.2910510105

[b20] Jensen-Jarolim E. . AllergoOncology: the role of IgE-mediated allergy in cancer. Allergy 63, 1255–1266, doi: 10.1111/j.1398-9995.2008.01768.x (2008). 18671772 PMC2999743

[b21] Sherman P. W., Holland E. & Sherman J. S. Allergies: their role in cancer prevention. Q Rev Biol 83, 339–362 (2008). 19143335 10.1086/592850

[b22] Markiewicz M. A. & Gajewski T. F. The immune system as anti-tumor sentinel: molecular requirements for an anti-tumor immune response. Crit Rev Oncog 10, 247–260 (1999). 10468184

[b23] Singer J. & Jensen-Jarolim E. IgE-based immunotherapy of cancer: challenges and chances. Allergy 69, 137–149, doi: 10.1111/all.12276 (2014). 24117861 PMC4022995

[b24] Karagiannis S. N. . Recombinant IgE antibodies for passive immunotherapy of solid tumours: from concept towards clinical application. Cancer Immunol Immunother 61, 1547–1564, doi: 10.1007/s00262-011-1162-8 (2012). 22139135 PMC11028906

[b25] Schlehofer B. . Primary brain tumours and specific serum immunoglobulin E: a case-control study nested in the European Prospective Investigation into Cancer and Nutrition cohort. Allergy 66, 1434–1441, doi: 10.1111/j.1398-9995.2011.02670.x (2011). 21726235

[b26] Roessler K., Dietrich W. & Kitz K. Expression of BCL-2 oncoprotein on tumor cells and tumor-infiltrating lymphocytes (TIL) in meningiomas. Neurosurgical review 22, 205–209 (1999). 10682928 10.1007/s101430050017

[b27] Domingues P. . Tumor infiltrating immune cells in gliomas and meningiomas. Brain Behav Immun 53, 1–15, doi: 10.1016/j.bbi.2015.07.019 (2016). 26216710

[b28] Yu J. S. . Intratumoral T cell subset ratios and Fas ligand expression on brain tumor endothelium. Journal of neuro-oncology 64, 55–61 (2003). 12952286 10.1007/BF02700020

[b29] Du Z. . Increased expression of the immune modulatory molecule PD-L1 (CD274) in anaplastic meningioma. Oncotarget 6, 4704–4716, doi: 10.18632/oncotarget.3082 (2015). 25609200 PMC4467109

[b30] Dong H. . Tumor-associated B7-H1 promotes T-cell apoptosis: a potential mechanism of immune evasion. Nature medicine 8, 793–800, doi: 10.1038/nm730 (2002). 12091876

[b31] Rajaraman P. . Occupation and risk of meningioma and acoustic neuroma in the United States. Am J Ind Med 45, 395–407, doi: 10.1002/ajim.10363 (2004). 15095422

[b32] Inskip P. D. . Sociodemographic indicators and risk of brain tumours. Int J Epidemiol 32, 225–233 (2003). 12714541 10.1093/ije/dyg051

[b33] Flint-Richter P., Mandelzweig L., Oberman B. & Sadetzki S. Possible interaction between ionizing radiation, smoking, and gender in the causation of meningioma. Neuro-oncology 13, 345–352, doi: 10.1093/neuonc/noq201 (2011). 21339193 PMC3064606

[b34] GA W. . The Newcastle-Ottawa Scale (NOS) for assessing the quality of nonrandomised studies in meta-analyses., http://www.ohri.ca/programs/clinical_epidemiology/oxford.asp January 37, 2015.

[b35] DerSimonian R. & Laird N. Meta-analysis in clinical trials. Control Clin Trials 7, 177–188 (1986). 3802833 10.1016/0197-2456(86)90046-2

